# Mechanisms Regulating the Dynamics of Photosynthesis Under Abiotic Stresses

**DOI:** 10.3389/fpls.2020.615942

**Published:** 2021-01-28

**Authors:** Izhar Muhammad, Abdullah Shalmani, Muhammad Ali, Qing-Hua Yang, Husain Ahmad, Feng Bai Li

**Affiliations:** ^1^State Key Laboratory of Crop Stress Biology for Arid Areas, College of Agronomy, Northwest A&F University, Yangling, China; ^2^State Key Laboratory of Crop Stress Biology for Arid Areas, College of Life Sciences, Northwest A&F University, Yangling, China; ^3^Department of Horticulture, Zhejiang University, Hangzhou, China; ^4^College of Horticulture, Northwest A&F University, Yangling, China

**Keywords:** photosynthetic machinery, chlorophyll, degradation, biosynthesis, abiotic stresses

## Abstract

Photosynthesis sustains plant life on earth and is indispensable for plant growth and development. Factors such as unfavorable environmental conditions, stress regulatory networks, and plant biochemical processes limits the photosynthetic efficiency of plants and thereby threaten food security worldwide. Although numerous physiological approaches have been used to assess the performance of key photosynthetic components and their stress responses, though, these approaches are not extensive enough and do not favor strategic improvement of photosynthesis under abiotic stresses. The decline in photosynthetic capacity of plants due to these stresses is directly associated with reduction in yield. Therefore, a detailed information of the plant responses and better understanding of the photosynthetic machinery could help in developing new crop plants with higher yield even under stressed environments. Interestingly, cracking of signaling and metabolic pathways, identification of some key regulatory elements, characterization of potential genes, and phytohormone responses to abiotic factors have advanced our knowledge related to photosynthesis. However, our understanding of dynamic modulation of photosynthesis under dramatically fluctuating natural environments remains limited. Here, we provide a detailed overview of the research conducted on photosynthesis to date, and highlight the abiotic stress factors (heat, salinity, drought, high light, and heavy metal) that limit the performance of the photosynthetic machinery. Further, we reviewed the role of transcription factor genes and various enzymes involved in the process of photosynthesis under abiotic stresses. Finally, we discussed the recent progress in the field of biodegradable compounds, such as chitosan and humic acid, and the effect of melatonin (bio-stimulant) on photosynthetic activity. Based on our gathered researched data set, the logical concept of photosynthetic regulation under abiotic stresses along with improvement strategies will expand and surely accelerate the development of stress tolerance mechanisms, wider adaptability, higher survival rate, and yield potential of plant species.

## Introduction

Although multiple physiological, biochemical, and molecular processes collaboratively define plant productivity, where the stable photosynthetic performance is thought to be essential for healthy plant growth and development (Ashraf and Harris, 2013; Gururani et al., 2015; Nguyen et al., 2018; Sharma et al., 2020). Photosynthesis is a remarkable process, which is not only confined to the leaves of green plants but also occurs in young developing embryos of aquatic and land plants as well as in bacteria (Taiz and Zeiger, 2010; Pan et al., 2012). Photosynthesis fuels a number of metabolic processes by triggering the process of conversion of light energy into chemical energy (Chen et al., 2018; Demmig-Adams et al., 2018). Chloroplast is the houses of both the light and dark reactions of photosynthesis, and is highly responsive to abiotic stresses, heavy metal toxicity, nutrient deficiency or toxicity, hypoxia or anoxia, ultraviolet (UV) radiation, light intensity fluctuations (Mu et al., 2016, 2017; Sharma et al., 2016a, 2019; Kaur et al., 2017; Demmig-Adams et al., 2018; Kohli et al., 2018; Paunov et al., 2018; Soares et al., 2018; Yadav et al., 2018). Abiotic stresses also have a negative impact on photosystem I (PSI) and PS II, electron transport chain (ETC), and chlorophyll (Chl) biosynthesis (Xia et al., 2006; Efeoglu and Terzioglu, 2009; Kalaji et al., 2016; Sharma et al., 2016b). Additionally, abiotic stresses reduce stomatal conductance, inducing oxidative stress, which decreases the activity of ribulose-1,5-bisphosphate carboxylase/oxygenase (Rubisco) and obstructs the process of photosynthesis (Allen and Ort, 2001; Crafts-Brandner and Salvucci, 2004; Chaves et al., 2009; Zhang et al., 2014; Kohli et al., 2017). Reactive oxygen species (ROS) are synthesized in various compartments including plastids, mitochondria, peroxisomes, and the apoplast via different pathways and is controlled by the ROS gene network (Mittler et al., 2004). In addition, each cellular compartment controls its own ROS homeostasis, where different ROS levels in various compartments generate a particular ROS signature. Further, different ROS signature resulted from unlike abiotic stresses and/or combination of different abiotic stresses are decoded via different ROS sensors and thereafter initiate stress-specific signal in the affecting plant. Previous reports stated that ROS production occurs in chloroplasts within the ETCs of PSI and PSII during light reactions, and is enhanced when carbon dioxide (CO_2_) supply is restricted and ATP synthesis is impaired (Takahashi and Murata, 2008; Nishiyama and Murata, 2014; Noctor et al., 2014; Tikkanen et al., 2014). The generation of ROS under normal conditions are the byproducts of metabolic pathways such as photosynthesis, respiration, and photorespiration, while over production of reactive oxygen species (ROS), such as hydrogen peroxide (H_2_O_2_), singlet oxygen (^1^O_2_), the superoxide (${\text{O}}_{2}^{-}$), and hydroxyl (HO^.^) radicals, is a general feature under abiotic stresses (Anjum et al., 2016a, 2017; Soares et al., 2016b; Guo et al., 2017a; Czarnocka and Karpiński, 2018; Kaur et al., 2019; Sharma et al., 2019). There are two major sources of ROS during abiotic stresses, metabolic ROS [generated as a consequence of disruptions in metabolic activity (Miller et al., 2010)] and signaling ROS [produced for signaling in response of the abiotic stress-response signal transduction network (Mittler et al., 2012; Tikkanen et al., 2014)]. The enhanced level of intracellular ROS within the cells are mitigated by ROS detoxifying proteins such as superoxide dismutase (SOD), ascorbate peroxidase (APX), catalase (CAT), glutathione peroxidase (GPX), and peroxiredoxin (PRX) (Mittler et al., 2004), and ascorbate or glutathione (GSH) that are present in almost all subcellular compartments (Takahashi and Murata, 2008; Schmutz et al., 2010; Upadhyaya et al., 2011). However, ROS-scavenging systems is not that much efficient to completely remove intracellular ROS, therefore to increase abiotic stress tolerance, genetic manipulation of antioxidants and ROS-scavenging enzymes may be more appropriate approach (Tóth et al., 2011).

Plants adopt several mechanisms for photoprotection, like photorespiration (Streb et al., 2005), cyclic electron flow (Huang et al., 2015b), alternative electron flow (Laureau et al., 2013), and antioxidant systems (Streb et al., 2003). Photorespiration consumes excess NADPH and, thus, alleviates its over-accumulation on the acceptor side of PSI, preventing the over-reduction of photosynthetic electron chains (Huang et al., 2015a). For example, the alpine herb, *Ranunculus glacialis*, excess electrons are transferred to oxygen when photorespiration is blocked, due to a high content of plastid terminal oxidase (PTOX). This PTOX has the capacity to transfer electrons from plastoquinone directly to the oxygen and, thus, avoid a reduction in the plastoquinone pool, thereby protecting the chloroplasts from over-reduction (Laureau et al., 2013). Moreover, PTOX can keep the plastoquinol pool oxidized under cold, heat, or high-light stresses, and thus alleviate photoinhibition of PSII.

Here, we review the impact of major abiotic stresses, such as drought, heat, salinity, and heavy metals, on photosynthetic machinery, especially in agricultural crops, and the role of different phytohormones, transcription factors, and key enzymes involved in photosynthetic reaction center under stress conditions. Additionally, we highlight the utility of beneficial/stimulant compounds that help to maintain the viability and activity of the photosynthetic system. Lastly, we discussed some future perspectives that can improve photosynthesis under stressful conditions. We believe that this review provides a comprehensive summary of the photosynthesis related research conducted to date and will be useful for crop improvement in the future.

## Major Factors Limiting Photosynthesis and Plant Growth

### Effects of High Temperature on the Efficiency of Photosynthesis

Global increase in temperature is currently one of the biggest problems affecting plant survival. Rise in temperature above a certain threshold level impairs cellular homeostasis and plant metabolism, decrease in plant growth, biomass, and final yield components (Dutta et al., 2009; Ashraf and Harris, 2013; Mathur et al., 2014; Ye et al., 2016; Sharma et al., 2020). Low or freezing temperature affects photosynthetic parameters such as stomatal conductance, carbon reduction cycle, transpiration rate, and thylakoid electron transport (TET) (Hou et al., 2016). Photosynthesis is highly sensitive to heat stress (Wang D. et al., 2010; Centritto et al., 2011), as high temperatures disrupt the thylakoid membrane and inhibit membrane-associated electron carriers and enzymes, thus decrease the rate of photosynthesis (Ristic et al., 2008; Rexroth et al., 2011). Although plants are capable of fine-tuning their photosynthetic ability in response to high temperatures, but short-term extreme temperatures disrupt Chl biosynthesis within plastids, leading to reduced Chl accumulation, while high temperature for a longer time trigger the process of Chl degradation and even can cause irreversible damages to Chl synthesis (Ristic et al., 2008; Efeoglu and Terzioglu, 2009; Balouchi, 2010; Reda and Mandoura, 2011; Rexroth et al., 2011; Antoniou et al., 2017). Additionally, the photosynthetic apparatus senses the heat stress and responds by diverting the cellular energy to the redox center (Biswal et al., 2011). The photosynthetic apparatus is very sensitive to heat stress, as where the site of inhibition even more quickly responds than cellular disruptions (Mathur et al., 2014). Rubisco is the key photosynthesis enzyme and the enzymatic activity of Rubisco swiftly decreases by thermal stress which thereafter affect the process of photosynthesis (Anjana and Allakhverdiev, 2017). In several species, Rubisco activase (RCA) protein with molecular masses of 41 kDa (β-isoform) and 47 kDa (α-isoform) are capable of activating Rubisco (Salvucci et al., 1987), but they also have physiological significance in thermal sensitivity under heat stress conditions (Crafts-Brandner et al., 1997; Crafts-Brandner and Salvucci, 2004). Previously, it has been reported in rice (Wang D. et al., 2010) and spinach (Crafts-Brandner et al., 1997; Kim and Portis, 2006) that α-isoform is more thermostable than the β-isoform, indicating that the α-RCA isoform have crucial role in photosynthetic acclimation under mild heat stress (*in vivo*), whereas the β-RCA isoform have shown significant role in maintaining Rubisco's initial activity during normal condition. Thus, the genetic basis of RCA gene regulation and expression of two isoforms may be helpful for understand the mechanism of optimization Rubisco activation under prevailing environmental conditions

Noticeably, high temperature also disrupts the water-oxidizing complex (WOC) and the structural and functional integrity of the PSII reaction center and light-harvesting complex (LHC) (Lípová et al., 2010).

In plants, it has been reported that the high temperature inhibits Chl biosynthesis by decreasing the activity of biosynthetic enzymes (Dutta et al., 2009; Reda and Mandoura, 2011). For example, under heat stress the celery (*Apium graveolens* L.) leaves, Chl biosynthesis declined because of the down-regulation of genes involved in Chl biosynthesis (Huang et al., 2017). Similarly, in barley (*Hordeum vulgare* L.) seedlings, Chl biosynthesis was inhibited by high temperature treatment for 4–8 h; this was probably the result of the activity of 5-aminolevulinate dehydratase (ALAD), an enzyme actively involved in the pyrrole biosynthetic pathway, or the inhibition of protochlorophyllide (Pchlide) biosynthesis (Mathur et al., 2014). Previously, similar findings were reported in wheat (*Triticum aestivum* L. cv. HD2329) seedlings, where the analogous effect of Pchlide biosynthesis resulted in identical outcomes (Tewari and Tripathy, 1998, 1999). Furthermore, in soybean (*Glycine max* L.), treatment with shift to high temperature (28–38°C) decreased the total Chl and Chl a contents by 18 and 7%, respectively, and the Chl a/Chl b ratio by 3% as well as the sucrose, sugar and leaf soluble sugar contents by 9, 47, and 36%, respectively (Tewari and Tripathy, 1998, 1999; Mohanty et al., 2006). In young seedlings of cucumber (*Cucumis sativus* L. cv. Poinsette), low (7°C) and high (42°C) temperatures caused irreversible damage to the photosynthetic apparatus, thus inhibiting plant growth (Tewari and Tripathy, 1998). In potato (*Solanum tuberosum* L.), the thylakoid membrane was stable under moderately high temperature (35–45°C); however, a slight thermal stress (35°C for 2 h) decreased the electron transport and damaged the permeability of the thylakoid membrane (Sharkey, 2005), suggesting that de-epoxidized xanthophylls and thylakoid membranes are inefficient against heat-induced stress.

High temperature stress is usually accompanied by light stress, and the spatiotemporal cycles of both stresses damage the photosynthetic machinery (Tikkanen et al., 2012). The process of photoinhibition is associated with the thermosensitivity of PSII to high temperature. Mainly two key factors affect electron transport: (1) increased permeability of thylakoid membranes at high temperature, which results damage to the efficiency of PSII and the LHC, and (2) dependency of PSII integrity on electron transport (Janka et al., 2013). A previous study showed that moderately high temperatures with low light intensity do not cause serious damage to PSII but obstruct the repair of PSII after stress (Evans, 2013). PSI is more stable than PSII. Moderate heat stress stimulates PSI activity, characterized by the increased thylakoid proton conductance and electron flow around the PSI, by producing higher ATP. Subsequently, high NADPH/ATP ratio reduces the plastoquinone (PQ) pool in stromal donors, which activates the NADH-mediated cyclic electron flow (Sharkey, 2005; Sharkey and Zhang, 2010).

In spinach (*Spinacia oleracea* L.), the intrinsic proteins of PSII cleaved the C-terminal end of the D1 protein under heat stress (40°C for 30 min), producing 9- and 23-kDa N-terminal fragments in thylakoids, and the slow repair process of the damaged D1 proteins reduced the cyclic electron flow under low light intensity, which negatively impacted plant growth and productivity (Yoshioka et al., 2006). Additionally, the FtsH protease, which localizes to the thylakoid stroma, translocated to the thylakoid granule to phosphorylate the D1 protein (Komayama et al., 2007). Here it can be assumed that D1 protein, degraded by heat and light stress (Nath et al., 2013).

**Figure 1:**
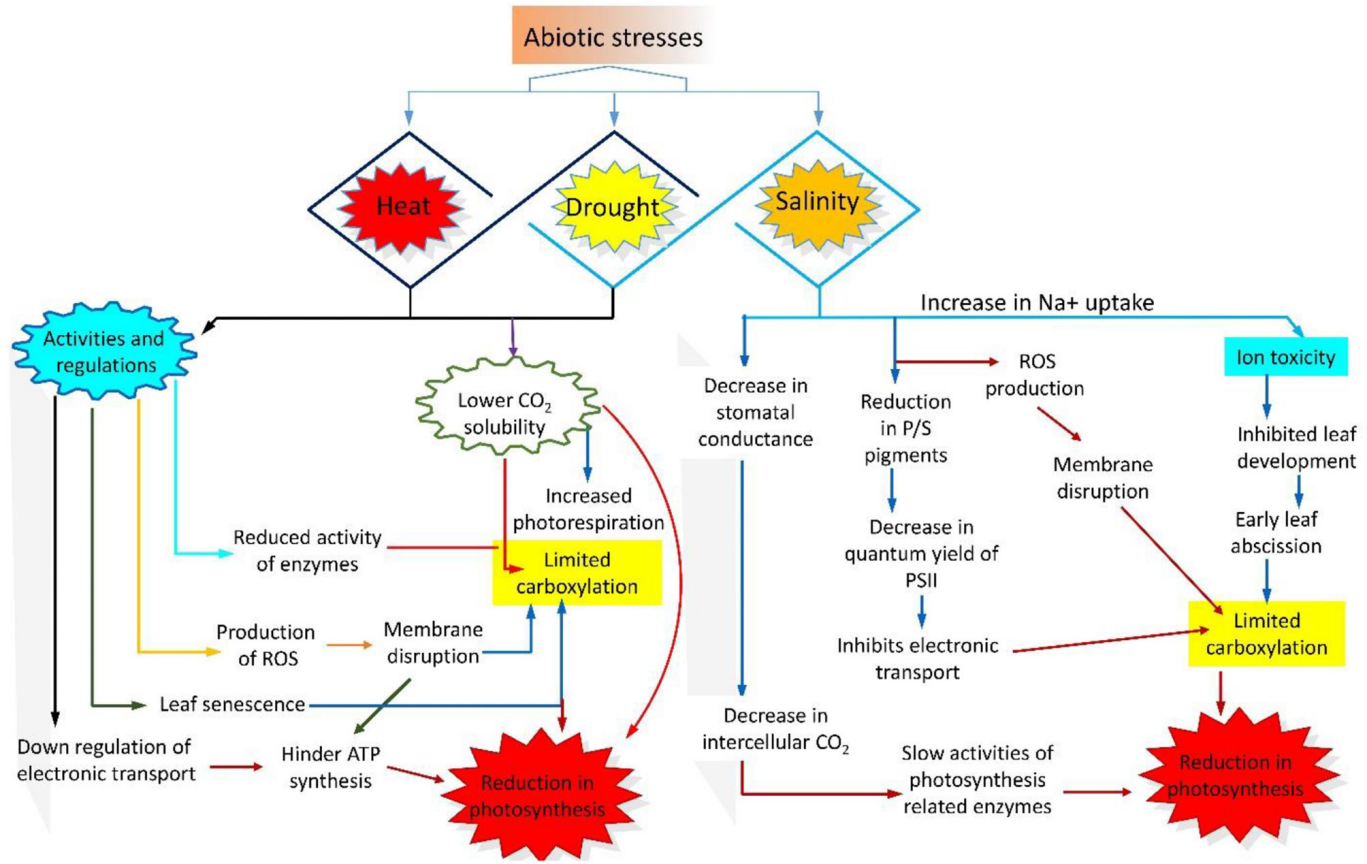
Schematic representation of the photosynthesis performance under abiotic stresses (heat, drought, and salinity). Drought and heat stress down-regulate enzymatic activity and electron transport chain (ETC) and cause membrane rupture, low CO_2_ solubility, leaf senescence, and reactive oxygen species (ROS) production. On the other hand, salinity causes ion toxicity, membrane disruption, reduced stomatal conductance, lower quantum yield of PSII, slow electron transport, and reduced activity of photosynthesis related enzymes.

Furthermore, the damage caused by heat stress in plants is attributed to the oxygen evolving complex (OEC), with alliance of cofactors in PSII and CO_2_ fixation by Rubisco, which mostly affects the potential of yield, additionally, some research identified that the functionless Rubisco decreases net photosynthesis under slight temperature stress (Sharkey, 2005; Velikova et al., 2012; Yamori et al., 2014). In very early studies, it was shown that Rubisco catalyzes two contending pathways (photosynthetic CO_2_ assimilation and photo-respiration), which are connected by the rate of oxygenase activity of Rubisco (Laing et al., 1974). Jordan and Ogren (1984) stated that the rate of photorespiration increases under high temperature, because of the relative specificity and relative solubility of CO_2_ compared with that of O_2_ (Jordan and Ogren, 1984). It has been reported that the lower expression of Rubisco per unit area under heat stress reduces the protein content in several species (Pérez et al., 2011). Although, plants grown in the natural environment generally exhibit great potential to tolerate high temperature, depending on the species, because of their adaptability and strong defense system. Figure 1 depicts the effect of heat stress on photosynthesis inhibition.

Moreover, an increase in the photosynthetic electron flux to oxygen (O_2_) may lead to excess production of superoxide radicals, hydrogen peroxide (H_2_O_2_) and other ROS that can damage proteins, lipids, and pigments (Li H. et al., 2017). Under low heat stress, ROS mainly disrupt the PSII repair system; however, it does not directly affect the PSII reaction center. Furthermore, the accumulation of compatible solutes, such as glycine betaine, in the vicinity of PSII membrane induces the expression of stress related proteins, and help in the stress damaged photosynthetic machinery (Evans, 2013). Thermal stress also induces changes in the total Chl and carotenoid contents of leaves, thus affecting the photoinhibition/photochemical intensity, resulting in reduced quantum yield of PSII (Fv/Fm), and enhanced peroxidation in the leaf cell membrane and decreased membrane thermostability, alter malondialdehyde (MDA) content, and electrolyte leakage (EL) (Cui et al., 2017; Zuo et al., 2017). These findings suggest that photosynthesis is sensitive to variation in temperature, and heat stress significantly affects the photosynthetic machinery, chlorophyll pigments, biosynthetic pathways, thus disturbing the overall morpho-physiology of higher plants. Identification of germplasm and development of transgenic lines with superior heat tolerance can be helpful to deal with high temperature stress.

### Salt Stress Markedly Affects Photosynthesis

Excess salt or saline soil substantially alters biochemical and physiological processes, especially during photosynthesis, causing stunted plant growth, and poor productivity. Salt stress accounts for ~50% reduction in crop productivity (Gururani et al., 2015; Ahmad et al., 2018; Sharma et al., 2020). Moreover, salinity-induced osmotic stress reduces photosynthesis via the ionic effect on the structure of subcellular organelles and the inhibition of metabolic processes (Lawlor, 2009; Sade et al., 2010; Ahmad et al., 2020). The cellular membranes exhibit stress responses (Ashraf and Ali, 2008; Tayefi-Nasrabadi et al., 2011), high concentration of ions, such as sodium (Na^+^) and chloride (Cl^−^) ions, in chloroplasts causes significant damage to the thylakoid membrane (Wu and Zou, 2009; Omoto et al., 2010). Furthermore, inorganic salts at high concentrations can cause irrecoverable inactivation of photophosphorylation and obstruction of electron transport in the thylakoid membrane (Veiga et al., 2007; Mittal et al., 2012).

Previously, several studies showed that severe salt stress breaks down Chl, and the excess sodium ions Na^+^ effect electron transport and destabilize photosynthetic activity (Pinheiro et al., 2008; Li et al., 2010). A reduction in photosynthetic pigments under salt stress was reported in several plant species such as wheat (Arfan et al., 2007; Perveen et al., 2010), alfalfa (*Medicago sativa*) (Winicov and Seemann, 1990), castor bean (*Ricinus communis*) (Pinheiro et al., 2008), and sunflower (*Helianthus annuus*) (Ashraf and Sultana, 2000; Akram M. S. and Ashraf, 2011). Najafpour et al. (2015) reported that the high Na^+^ ion concentration in cells alters the potassium ion (K^+^): Na^+^ ratio, which instantaneously affects the bioenergetic processes of photosynthesis (degradation of photosynthetic pigments) in cyanobacteria as well as in plants (Najafpour et al., 2015). Similarly, Eckardt (2009) showed that salt-induced alterations impair the biosynthesis and accelerate the degradation of photosynthetic pigments (Eckardt, 2009). Other studies further summarized the reduction of Chl a and Chl b contents under salt stress in field crops, such as *Paspalum vaginatum* (Ivanov and Velitchkova, 2014), *Centaurium erythraea* (Sundby and Andersson, 1985), common bean (*Phaseolus vulgaris*) (Sundby and Andersson, 1985), *Catharanthus roseus*, cowpea (*Vigna unguiculata)* (Taffouo et al., 2010), and *Vigna subterranean* (Muranaka et al., 2002).

Additionally, under salt stress, the Chl precursors, glutamate, and 5-aminolaevulinic acid (ALA), remarkably affect the biosynthesis of Chl in sunflower callus and plants (Vieira Santos et al., 2001; Santos, 2004). Salt tolerance plant species show an increase in Chl content, when grown under saline conditions (Khan et al., 2009; Akram N. A. and Ashraf, 2011), This lead to the concept that salt tolerant plant species with high Chl content exhibit greater membrane stability and higher Chl pigment content. So far, several salt tolerant plant species such as pea (*Pisum sativum*) (Noreen et al., 2010), melon (*Cucumis melo*) (Romero et al., 1997), sunflower (Akram N. A. and Ashraf, 2011), wheat (Raza et al., 2006; Arfan et al., 2007), alfalfa (Monirifar and Barghi, 2009), and proso millet (*Panicum miliaceum*) (Sabir et al., 2009) have been screened for their salt tolerance capacity. In contradiction to the aforementioned salt screening strategy, Juan et al. (2005) observed weak linkage between leaf Na^+^ level and photosynthetic pigment content in tomato (*Solanum lycopersicum*) plants, indicating that chlorophyll content assimilation is not always associated with salt tolerance, but is an indicator of saline conditions, depending on the plant species (Juan et al., 2005).

A recent study revealed that salt stress (7–8 dS m^−1^) is also responsible for the reduction in the amount of carotenoids and Chl in sugarcane (*Saccharum officinarum* L.) plants at different growth stages (Gomathi and Rakkiyapan, 2011). Another study in hot pepper (*Capsicum annuum* L.) showed a significant increase in Chl and carotenoid contents in the presence of 60 mM salt (Ziaf et al., 2009). Therefore, we speculate that the carotenoid content of plants under salt stress could be a useful selection criterion. Additionally, salt tolerance at gene level has great potential; for example, the rice (*Oryza sativa* L.) *OsSUV3* gene, which encodes the Ski2 family of DExH/D-box helicases, functions under salt stress to facilitate photosynthetic processes and assist the antioxidant machinery (Tutej et al., 2014). Together, the studies described above prove that Chl content, photosynthetic pigments, membrane damage, and biochemical changes are of the primary targets under salt stress, where membrane instability and pigment degradation severely affect the growth, development, and physiological parameters of plants (Figure 1).

### Regulation of the Photosynthetic Machinery Under Drought Stress

Drought stress is one of the most crucial environmental factor impairing photosynthesis and thereby limiting plant growth and yield (Donohue et al., 2013; Hui et al., 2018; Tanveer et al., 2019). Water deficiency limits the efficacy of the photosynthetic apparatus, causes substantial damage to the thylakoid membrane and reduces the Chl content (Din et al., 2011; Smolikova et al., 2017; Demmig-Adams et al., 2018). Figure 1 links the photosynthesis-limiting drought stress with other abiotic stresses in the flow chart. Global warming and lower availability of underground water promote the occurrence of drought worldwide, thus affecting plant growth and productivity. To ensure survival under such a harsh environment, plants recruit their defense system and ultimately adjust themselves by adopting different strategies, such as stomata closure (to decrease transpiration), osmotic adjustment, and enhanced tolerance level (Zhang, 2007; Sharma et al., 2019). Xerophytes are an excellent example of such plants. Under drought stress, xerophytes absorb more water, reduce the transpiration rate, and exhibit morphological changes, such as a thick cuticle layer and stomatal closure (Macková et al., 2013). Beside that some non-stomatal mechanisms also decrease photosynthesis (Hajiboland et al., 2017); for example, the intake of CO_2_ is disturbed by stomatal closure, which alters enzymatic activities, causes membrane disruption and reduces ATP synthesis and ribulose-1,5-bisphophate (RuBP) regeneration, thus inhibiting RUBISCO activity and effect the process of photosynthesis. Additionally, mild drought stress usually inhibits photosynthesis and stomatal conductance (Medrano et al., 2002), usually plants in such situation adopt defensive strategy by increasing the water use efficiency (WUE) by controlling net CO_2_ and transpiration rate in leaf tissues (Chaves et al., 2009). By contrast, under severe drought stress, dehydration of mesophyll cells allows the utilization of available CO_2_, which remarkably inhibits the metabolic processes of photosynthesis, leading to reduction in WUE and root hydraulic conductivity (Karaba et al., 2007; Dias and Brüggemann, 2010; Anjum et al., 2011; Damayanthi et al., 2011; Din et al., 2011).

Additionally, drought exerts a negative effect on the PSII by reducing its quantum yield (Albert et al., 2011; Tattini et al., 2014). A study disclosed that during drought stress, the turgor pressure of cells decreased, which reduced the shoot length, biomass, and plant growth (Semerci et al., 2017). The photosynthetic machinery is significantly affected by water-deficit condition (Sun et al., 2013); for example, water deficiency results in the degradation of the thylakoid membrane and Chl pigments and decreases the Chl content (Bertioli et al., 2016). Moreover, altered Chl fluorescence kinetics affects PSII (Zhang et al., 2011). This was confirmed by Batra et al. (2014); where the reduced Chl fluorescence and water content, creates dehydration, and further targeting PSII electronic transport and PQ reduction under water-deficit condition (Batra et al., 2014).

Teixeira et al. (2016) reported that the Chl content of soybean seeds under transient drought stress had showed effects on nutritional value and oil quality (Teixeira et al., 2016). Additionally, the impaired expression of SGR (STAY-GREEN), a chloroplast targeted protein that act as key regulator of Chl degradation and NYC1 (NON-YELLOW COLORING 1) under heat and drought stress inhibit the process of chlorophyll degradation and retention in green soybean seeds. In wheat, the relative contribution of ear (spike and awns) to grain filling is severely affected by drought stress, for instance the CO_2_ assimilation is reduced due drought and eventually disturb the rate of photosynthesis (Kottmann et al., 2014; Merah et al., 2018). Ashraf and Harris (2013) reported that Chl content assimilation does not show a positive correlation with drought condition in wheat plants, suggesting that it may be due to variation in Chl synthesis among the cultivars mediated by the alteration in the activities of specific enzymes involved in the biosynthesis of Chl (Ashraf and Harris, 2013). Despite the above contradiction in the assimilation of Chl content, degradation, and biosynthesis under drought stress has been reported by various researchers; for example, in some genotypes of black gram (*Vigna mungo*), Hamada and Al-Hakimi (2001) and Pirzad et al. (2011) reported an imbalance in accumulation of Chl (Hamada and Al-Hakimi, 2001; Pirzad et al., 2011). However, the regulation of enzymatic activity during Chl biosynthesis is dependent on the response of a particular genotype. Additionally, enzymatic activity of chlorophyllase and peroxidase is involved in the rapid breakdown of Chl that reduce the process of synthesis (Kaewsuksaeng, 2011). Moreover, a greater amount of Chl b than that of Chl a under drought condition has been reported by Jaleel et al. (2009) and Jain et al. (2010). In *Brassica* and wheat, the Chl a/b ratio was highly reduced in susceptible cultivars under drought stress, but was slightly increased in tolerant genotypes (Ashraf and Mehmood, 1990; Ashraf, 1994). In rice, helicase domain-containing proteins have shown up-regulation in response to drought stress and functions as maintaining photosynthesis and antioxidant machinery (Ambavaram et al., 2014; Chintakovid et al., 2017), Moreover, the genes encoding GAPDH and FNR [the key enzymes influencing NADP(H) homeostasis are affected by osmotic-stress treatments], suggests that drought tolerance in rice may be mediated by photosynthesis-related adaptations by utilizing the NADP(H) homeostasis.

### Effect of Heavy Metal Pollution on the Photosynthetic Machinery

**Figure 2:**
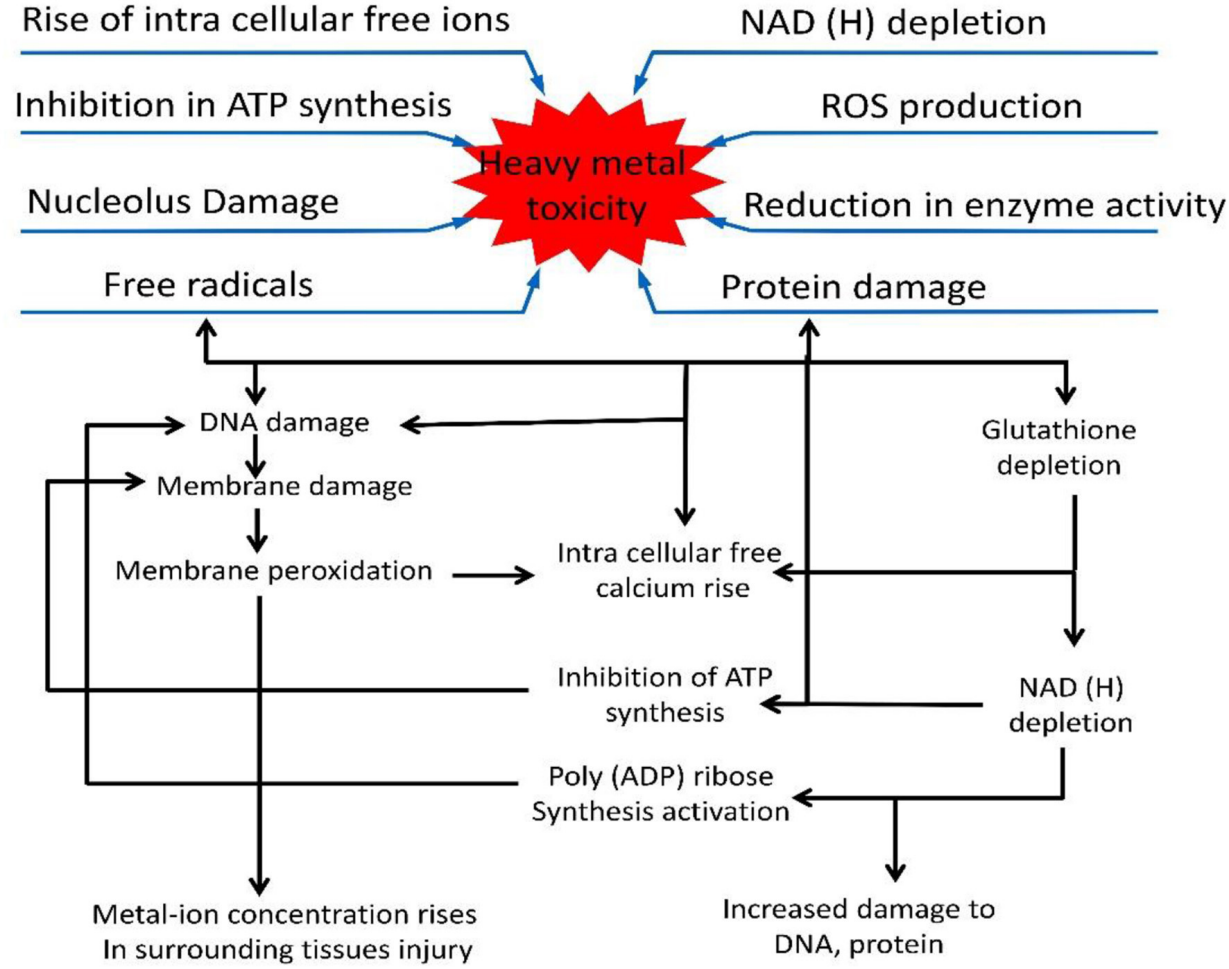
Diagram showing the general and specific effects of heavy metals on plants. Heavy metals affect ROS production, inhibit ATP synthesis and damage DNA and proteins. The damage to DNA and proteins is shown separately. Free radicals damage DNA and cause membrane peroxidation when metal ions surround the site of tissue injury, causing ATP inhibition, and NADH depletion.

Soil contamination with heavy metals such as cadmium (Cd), copper (Cu), zinc (Zn), nickel (Ni), cobalt (Co), chromium (Cr), lead (Pb), and arsenic (As) is generally caused by the application of phosphate-rich fertilizers, sewage sludge, industrial waste, wind-blown dust, incinerator emissions, traffic, volcanoes, and hard water practices (Bagur et al., 2009; Ghori et al., 2019). Heavy metal pollution considerably inhibits plant growth by causing Chl degradation, DNA and protein damage as well as enzymatic inhibition (Figure 2). When coupled with other environmental stresses, heavy metal pollution causes more severe damage (Srivastava et al., 2012; Kumar et al., 2019; Sharma et al., 2020). Abiotic stresses like heavy metals induce excessive accumulation of ROS and cause oxidative stress in plants (Li et al., 2016). Interestingly, metallothioneins (MTs) have been proposed an alternative tool by which plants protect themselves from stress-induced oxidative damage (Yu et al., 2019). Hassinen et al. (2011) reported the role of MTs in abiotic stress tolerance as ROS scavengers, though the mechanisms through which MTs mediate ROS homeostasis remain unclear (Hassinen et al., 2011).

Moreover, heavy metal stress considerably alters the biological, biochemical and metabolic processes of plants (Anjum et al., 2015, 2016a,b,c; Handa et al., 2018; Shahzad et al., 2018a; Khanna et al., 2019; Kohli et al., 2020), and alteration at the cellular and molecular levels causes severe damage, thus blocking the functional group and active site of enzymes, thereby disrupting membrane stability and transcriptional regulation (Rascio and Navari-Izzo, 2011; Guo et al., 2016, 2017b). Metal ion toxicity causes DNA damage and/or impairs DNA repair mechanisms, disrupts membrane functional integrity, affects enzymatic activity, and perturbs protein function (Tamás et al., 2014; Figure 2).

LHCII (light harvesting complex II) is the basic pigment-protein complex of PSII, which harvests light energy and converts it into chemical energy. This protein complex plays a protective role by dissipating excess light energy and efficiently channelize excitation energy (Barros et al., 2009). Cd stress affects the LHCII (Parmar et al., 2013). In rye (*Secale cereale*), Cd stress reduced the dissipation of excitation energy, indicating that Cd stress either altered the quenching center (QC) or interfered with energy transfer between proteins and pigments (Janik et al., 2010). Ahmed and Tajmir-Riahi (1993) confirmed changes in LHCII by Pb, where imperfect assembly of its components triggered disintegration (Ahmed and Tajmir-Riahi, 1993).

**Figure 3:**
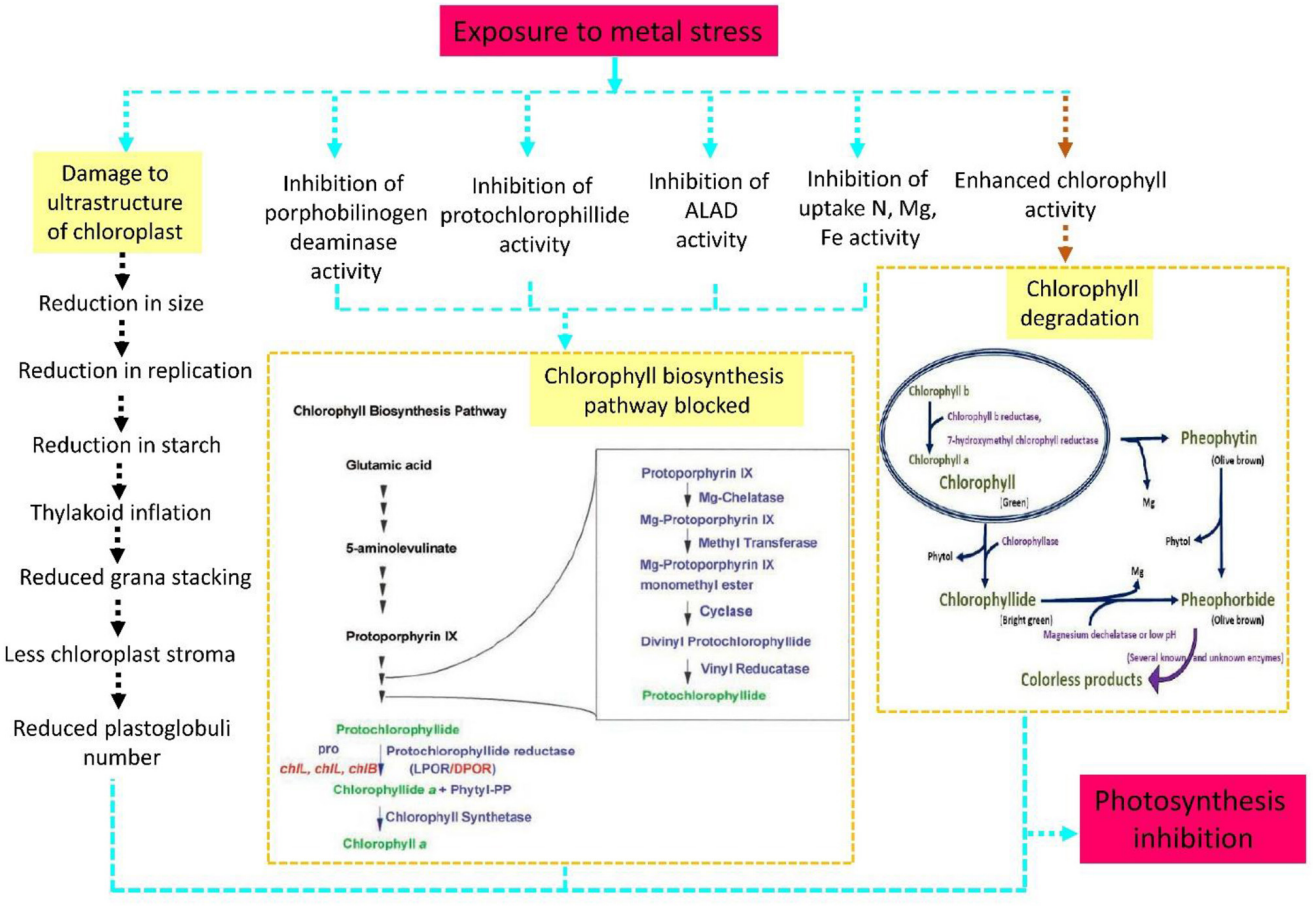
Heavy metal stress damages the chloroplast structure and chlorophyll (Chl) biosynthesis and degradation processes during photosynthesis. Toxicity due to metal ions gradually affects enzymatic activity and inhibits Chl components and the uptake of essential elements, finally blocking the Chl biosynthesis pathway. The blue arrow depicts this process in a step-by-step manner.

Plants grown in metal contaminated soils exhibit leaf chlorosis due to the reduced chloroplast size (Shahzad et al., 2016, 2017). The ultra-structure of chloroplast is greatly affected by metal ions (Figure 3). For example, Cd, a potent inhibitor of photosynthesis, alters the chloroplast shape, decreases chloroplast size, destroys Chl, reduces starch accumulation and expands the thylakoids (Najeeb et al., 2011; Parmar et al., 2013; Kapoor et al., 2019). Other metals such as Cr decrease the absorption of magnesium (Mg) and nitrogen (N), consequently reducing the Chl content (Singh et al., 2013). Pb toxicity accelerates Chl degradation by increasing the activity of chlorophyllase (Drazkiewicz, 1994). High levels of Ni affect the photosynthetic apparatus and inhibit the synthesis of pigments (Soares et al., 2016a, 2019; Shahzad et al., 2018a,b). Additionally, Ni stress alter the composition of lipid membrane and disturb the activity of chlorophyll molecule and Rubisco (ribulose-1,5-bisphosphate carboxylase oxygenase) (Kohli et al., 2020).

Early studies established that the photosynthetic apparatus, Chl and carotenoid concentrations and plant growth were greatly decreased under Ni and UV-B combination stress. It is possible that Mg in Chl is replaced by Ni, which destroys Chl and damages thylakoid membranes in cabbage leaves and wheat shoots, respectively (Molas, 2002; Gajewska et al., 2006). Similarly, the combined effect of heavy metals and drought stress can cause deleterious effects on Chl content in red maple and effect the xylem structure and hydraulic conductivity (De Silva et al., 2012).

Transition metals (Cu, Zn, manganese [Mn], and iron [Fe]) play critical roles in physiological processes of all living organisms, such as Cu is essential for respiration, photosynthesis, cell wall integrity, ethylene perception, and ROS metabolism in plants (Burkhead et al., 2009). Excess amounts of transition metals leads of Chl degradation by Chl-degrading enzymes, thereby increasing the sensitivity of PSII to light (Pätsikkä et al., 2002). Cu, Hg, Ni, Zn, and aluminum (Al) metal ions interact with three extrinsic polypeptides, situated in the lumen of the thylakoid membrane, additionally, intrinsic proteins (inner antenna protein), are released from the PSII reaction center under Cu toxicity (Sigfridsson et al., 2004; Boisvert et al., 2007). At optimum concentrations, Zn plays an important role in plant growth; however, excess Zn significantly reduces the synthesis of photosynthetic pigments and negatively impacts photosynthesis (Paunov et al., 2018). Zn and Cd stresses exert a synergistic effect by increasing the oxidative stress and restoring the Chl content (Cherif et al., 2011). Therefore, we speculate that transition metals in combination may reduce or restore the damage caused to the photosynthetic machinery for a short period of time under specific conditions. Comparative analysis of Cd and Zn stresses in tobacco (*Nicotiana benthamiana*) leaves revealed that under Cd stress, key enzymes involved in Chl biosynthesis were significantly down-regulated, decreasing the Chl content, expression of PSII (donor, receptor and core side) proteins and photosynthesis (Zhang et al., 2020). Several studies have reported the harmful effects of Cd and Zn on photosynthesis, thylakoid membrane ultra-structure, photosynthetic pigments, Chl fluorescence, electron transport, light capture, dark respiration, stomatal conductance, and Calvin cycle enzymes (Krupa, 1999; Vassilev et al., 2011; Paunov et al., 2018). Furthermore, both metals considerably reduce the activity of PSII and to some extent that of PSI as well as the rate of photosynthetic electron transport (Krupa, 1999; Vassilev et al., 2004).

### Impact of Light Intensity on Photosynthesis

Light is the key factor responsible for the healthy growth and development of plants, and fluctuation in light intensity negatively affect plant physiology and biochemistry and inhibit the process of photosynthesis (Demmig-Adams and Adams, 1992; Berenschot and Quecini, 2014; Wang Y. et al., 2017; Sharma et al., 2020). For instance, high light intensity generates harmful oxygen radicals (Gururani et al., 2015) and disrupt LHC (light harvesting complex), thus causing photoinhibition (Erickson et al., 2015), whereas insufficient light reduces the photosynthetic efficiency as well as CO_2_ and N metabolism and boosts oxidative stress (Solymosi and Schoefs, 2010; Wang et al., 2013). Additionally, low light significantly reduces photosynthesis and the efficiency of stomatal conductance, resulting in a rapid increase in the intercellular CO_2_ concentration in leaves (Liu et al., 2014). Thus, low light stress inhibits the photosynthetic machinery, stomatal conductance, maximum quantum efficiency of PSII, transpiration rate, WUE, and net photosynthesis rate (Pan and Guo, 2016; Zhang et al., 2016). Previous reports described that photosynthetic organisms are frequently exposed to high light intensities, in order to avoid photo-oxidative stress, plants adopt a photo protective mechanism by inducing non-photochemical quenching (NPQ) for safe dissipation of the excess energy as heat (Demmig-Adams and Adams, 1992; Erickson et al., 2015; Tibiletti et al., 2016).

Phytochromes and cryptochromes are multiple sensory photoreceptors that respond to light signals. However, the mechanistic explanation of these phytochromes is not yet fully understood. The function of direct protein–protein interaction, SPA1/COP1 E3, ubiquitin ligase complex, bHLH transcription factors, and transcription regulation of BIC genes regulates the photoreceptor coactions, which may serve as a safe valve to prevent cryptochromes from over-reacting in germinating seedlings (Wang Y. et al., 2017; Wang et al., 2018). Photoinhibition is caused by the functional failure of the PSII reaction center (Gururani et al., 2015), where poor oxidation during high light intensity damages the intrinsic key responsible D1 protein (Chen et al., 2012) and degradation in PSII reaction center become photo-inactivated. Additionally, high light intensity also reduces mitochondrial activity, photochemical efficiency as well as the dissipation of excess light energy in the form of heat, thus damaging PSI and PSII (Faseela and Puthur, 2018). Additionally, the quantum efficiency of PSII is hindered by electron transport, photochemical efficacy, photo-oxidation under high light stress accompanied by high temperature (Faseela and Puthur, 2017). Furthermore, high light intensity induces Chl b degradation by the Chl b reductase enzyme (Sato et al., 2015), although plants have the ability to modify their physiology by altering processes such as stomatal conductance and transpiration (Cowie et al., 2016).

Severe light stress also causes photodamage in the photosynthetic apparatus. For example, in tropical regions, plants face high light intensity with high temperature, which causes photon excitation in the chloroplast and reduction of photochemical efficiency (Elsheery and Cao, 2008). On the other hand, leaves of plants grown in shade absorb excess light energy, leading to photoinhibition (Krause et al., 2004; Takahashi et al., 2009). Additionally, the efficiency of PSII also decreases when the upper leaves are growing in shade, although the performance of PSI is not significantly affected under these conditions (Barth et al., 2001; Krause et al., 2004). The sensitivity of PSI to photoinhibition was also observed in *Arabidopsis thaliana* and cucumber during chilling (Zhang and Scheller, 2004). Xu et al. (2016b) observed that high light intensity (1,000 μmol photons m^−2^ s^−1^) causes photodamage in the cells of *Dunaliella salina*, while free hydroxyl radicals induce PSI photoinhibition via oxidation (Sonoike, 2011; Xu et al., 2016b).

ROS production during photosynthesis is regulated by the interaction of O_2_ with the photosynthetic electron transport chain (ETC) (Li et al., 2009). Under excess light, ROS production increases, and to avoid the accumulation of ROS to harmful levels, plants employ antioxidant enzymes, detoxification, and repair mechanisms (Falk and Munné-Bosch, 2010; Pospíšil, 2014). Additionally, the production of ROS by high light intensity induces plant cell death (González-Pérez et al., 2011). Plastoquinone-9, a prenyl lipid that serves as an electron carrier between PSII and PSI, protects the photosynthetic apparatus by controlling photoinhibition of PSII under severe light stress (Ksas et al., 2015).

### Effect of Phytohormone Signaling Networks on Photosynthesis Under Stress

The interaction of plant hormones and cellular redox is crucial for the process of photosynthesis under different abiotic stresses (Mayzlish-Gati et al., 2010; Kim et al., 2012; Krumova et al., 2013). Previously, it was evident that impaired photosynthetic light harvesting in *Arabidopsis* mutants has strong interaction between the control of excitation energy transfer and hormonal regulation (Tikkanen et al., 2014). The metabolism of phytohormone regulation network by ROS generation may intricate to complex hormonal crosstalk in response to stressful conditions presented in Table 1.

**Table 1:** Effects of plant hormones on the photosynthetic machinery under normal and stress conditions in different plant species.

**Phytohormone**	**Species**	**Effect on photosynthesis related parameters**
Abscisic acid (ABA)	Arabidopsis	Down-regulates the expression of photosynthesis related genes (Staneloni et al., 2008; Xu et al., 2012)
		Regulation of *LHCB* genes by ABA mediation (Voigt et al., 2010; Xu et al., 2012; Liu et al., 2013)
	Rice and cabbage	Greater PSII efficacy, NPQ and PSII photochemistry and protection against salt and light-induced damages (Zhu et al., 2011)
Strigolactone (SL)	Arabidopsis	Regulation of *LHCB* genes, reduced activity of Rubisco, PSI and PSII and sensitivity to photosynthesis by GR24, a synthetic SL (Mashiguchi et al., 2009; Mayzlish-Gati et al., 2010)
Gibberellin (GA)	*Brassica napus*	Decreased GA activity in transgenic plants and improved Chl content and photosynthesis (Zhou et al., 2011)
	Citrange	Positive regulation of photosynthesis related genes (Huerta et al., 2008)
Ethylene (ET)	Mustard	Facilitate the functioning of PSII and Rubisco when exposed to heavy metal (Ni and Zn) stress (Khan and Khan, 2014)
Jasmonic acid (JA)	Arabidopsis	Improve efficiency of quantum of PSII, deprive photosynthesis (Attaran et al., 2014)
Salicylic acid (SA)	Wheat	Elevated PSII capacity and delayed but enhanced recovery of the damaged D1 protein under heat and high light intensity (Zhao et al., 2011)
Cytokinin (CK)	Tobacco	Improved transcriptional regulation of genes linked with PSII, Cytb6f complex, PSI, NADH oxidoreductase and ATP synthase complex (Rivero et al., 2010)
	Arabidopsis	Condensed quantum efficiency (QE) of PSII, damage D1 protein under high light intensity (Cortleven et al., 2014)

Strigolactones (SLs) play an important role in the regulation of genes associated with harvesting light. Synthetic SL compounds regulate several genes encoding LHC proteins, CAB proteins, and PSI and PSII components under stress conditions (Mayzlish-Gati et al., 2010). For example, in the Arabidopsis SL signaling mutant *max2*, the response to dehydration is down-regulated because of the suppression of genes involved in photosynthesis, suggesting that the association between the misregulation of genes involved in photosynthesis reduced drought tolerance and sensitivity to the high energy demands of photosynthesis in *max2* plants (Ha et al., 2014).

Gibberellins (GAs) regulates photosynthesis and promote seed germination and cell division (Huerta et al., 2008; Zhou et al., 2011). In cucumber cotyledons, GA and kinetin influence the functional site of PSI and PSII reaction centers, thereby encouraging the development of the photosynthetic electron transport system (Pedhadiya et al., 1987). Similarly, in broad bean protoplasts, short-term GA-3 treatment increased the net photosynthetic rate and O_2_ evolution (Yuan and Xu, 2001). In transgenic *Brassica napus* plants, the photosynthetic capacity increased with the decrease in GA bioactivity (Zhou et al., 2011). Higher levels of endogenous GA remarkably up-regulate genes involved in photosynthesis and drought tolerance (Huerta et al., 2008). In wild-type and transgenic Arabidopsis plants, GA-3 treatment activated GA-responsive genes and enhanced tolerance to heat, salt and oxidative stresses (Alonso-Ramírez et al., 2009). However, further studies are required to exploit the relationship between photosynthesis and endogenous/exogenous GA-3 levels under various abiotic stresses.

Brassinosteroids (BRs) play important roles in plant growth and development, abiotic stress responses and defense mechanism. BRs also influence the efficiency of PSII and photosynthetic CO_2_ fixation in land plants (Oh et al., 2010; Choudhary et al., 2012; Krumova et al., 2013). Previous reports uncovered the relationship between BRs and photosynthesis related genes in several plant species (Oh et al., 2011; Bai et al., 2012). For instance, in the Arabidopsis *brassinosteroid-insensitive1* (*bri1*) mutant, genes involved in the regulation of photosynthesis were significantly down-regulated, which reduced plant growth and photosynthetic activity (Kim et al., 2012). Moreover, further analysis revealed that Arabidopsis mutants with altered BR responses exhibit drastic changes in thylakoids, inhibition of O_2_ evolution, reduction in PSII quantum yield and smaller PSII complex (Krumova et al., 2013). The BR-induced changes in the thylakoid structure and regulation of PSII during photosynthesis have also been described in other studies (Dobrikova et al., 2014; Rothová et al., 2014). Although BR deficiency boosts the content of Chl and photosynthetic proteins in plants, changing the leaf color to dark green (Komatsu et al., 2010), exogenous BR treatment in pepper (*Capsicum annuum*) resulted in harmful effects on photosynthesis under drought stress by decreasing light use efficiency and non-photochemical quenching (NPQ) in PSII antennae (Hu et al., 2013). Based on the abovementioned studies, it can be concluded that intensive investigation should be practiced to describe the specific role of BRs in the PSII damage repair system and ameliorating changes in the thylakoid structure during the process of photosynthesis.

The most widely studied hormone, abscisic acid (ABA), plays a dynamic role in response in plants during abiotic stresses. ABA directly regulates the PSII-associated O_2_ evolution and granular chloroplast structure in plants (Maslenkova et al., 1989). Exogenous supply of ABA enhanced the amount of Chl, total carotenoids, and xanthophylls in leaves, and also help in excessive excitation energy on PSII (Barickman et al., 2014). In barley seedlings, ABA treatment significantly increased the photosynthetic apparatus under heat stress, although heat shock reduced the damage to the initial chloroplast fluorescence (Ivanov et al., 1992). ABA treatment up-regulates the expression of *LHCB* gene family, which is mainly involved in the adaptation to abiotic stress (Liu et al., 2013). Additionally, down-regulation of *LHCB* genes decreased ABA signaling, suggesting the involvement of ABA signaling in drought stress and ROS modulation (Xu et al., 2012). The inconsistency concerning the role of ABA in photosynthesis is possibly due to the dissimilar experimental system/methodology and the photosynthesis data obtained using different methods.

Salicylic acid (SA), a phenolic compound, is extensively involved in the process of plant growth regulation, physiology and biochemical activities of cells as well as in the response to stress conditions (Kunihiro et al., 2011; Drzewiecka et al., 2012; Li Z. et al., 2017). Therefore, it is important to discover the role of SA related genes in photosynthesis during abiotic stresses. Investigation in *Phillyrea angustifolia* plants under drought stress showed increased assimilation of endogenous SA and significantly decreased Fv/Fm in leaves (Munné-Bosch and Peñuelas, 2003). SA treatment improved the photosynthetic capacity of wheat plants (Arfan et al., 2007). Similarly, SA treated wheat leaves showed an improvement in Fv/Fm, photochemical activity of PSII, photosynthetic rate and electron transport, which reduced the damage caused by heat and high light intensity to the D1 protein and PSII (Zhou et al., 2011). Therefore, it can be established that SA pretreatment is associated with the chloroplastic heat shock proteins (HSPs), thereby up-regulating the photosynthetic rate. In other crops such as barley, exogenous SA treatment facilitated the adaptation to salt stress and improved the cell membrane integrity (El-Tayeb, 2005). In grapevine (*Vitis vinifera*) leaves, SA treatment under heat stress enhanced the PSII system parameters and net photosynthetic rate (Wang L. J. et al., 2010).

To date, only a few studies have described the roles of methyl jasmonate (MeJA) and ethylene in photosynthesis, and the regulatory mechanisms that provide stability to PSI and PSII under abiotic stresses remain unclear. However, exogenous application of ethylene demonstrated its role in the regulation of tolerance to Ni- and Zn-induced heavy metal stress by improving photosynthetic efficiency (Khan and Khan, 2014). Additionally, in sunflower, ethylene treatment increased the net photosynthetic rate and reduced the effects of excess Cu, thereby stabilizing the Fv/Fm ratio (Ouzounidou and Ilias, 2005).

**Figure 4:**
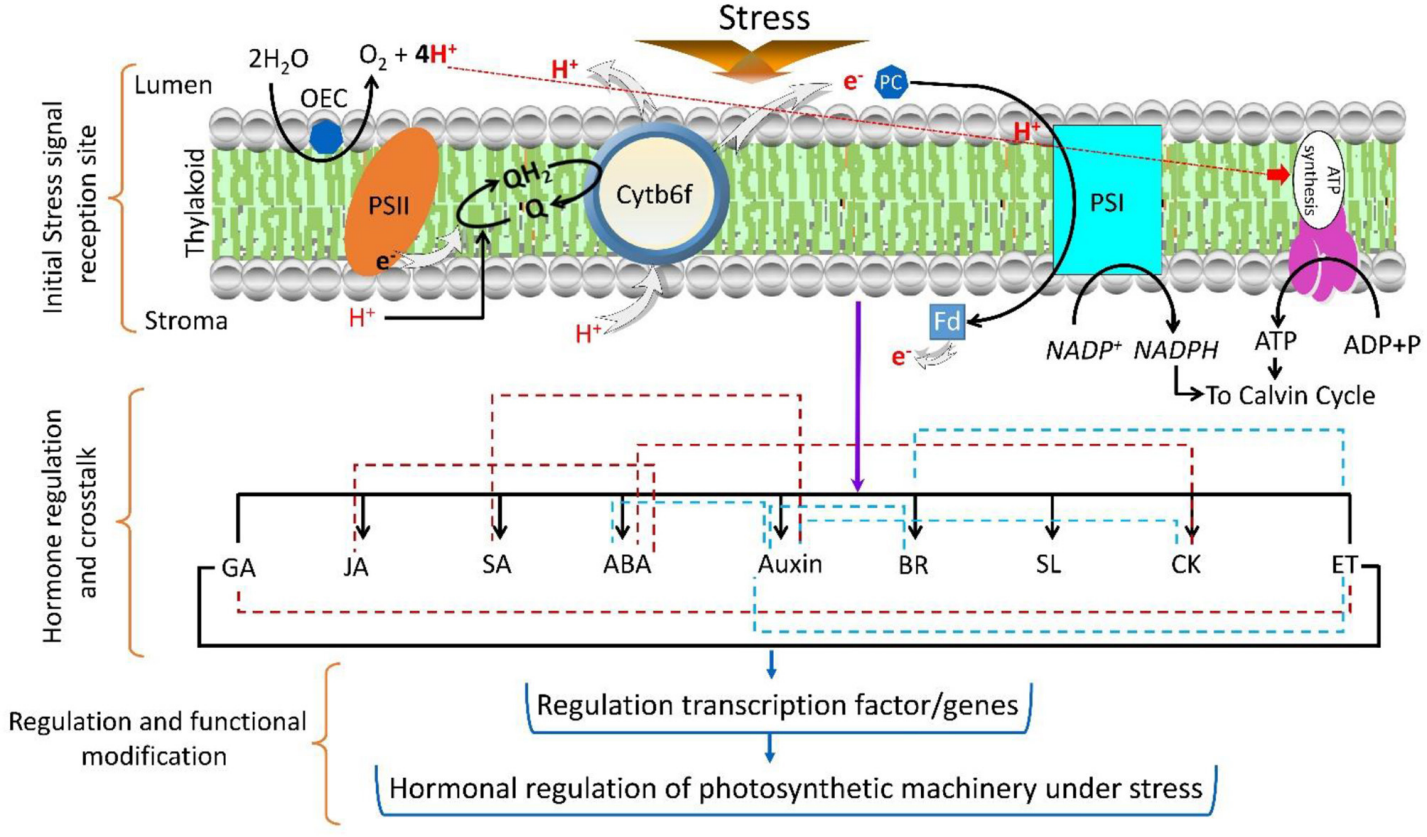
Regulation of the complex phytohormone network under stress conditions. The imposition of stress on the photosystem reaction center induces the hormonal signal transduction. The red dotted lines indicate the regulation of two hormone inhibitions, and green lines indicate the co-regulation of two hormones. The regulation of gene transcription by different hormones shows the involvement of the photosynthetic machinery.

Selenium (Se) guards the photosynthetic activity of *B. napus* seedlings under Cd stress, as high Cd-stressed plants may act as a trap for free radicals stabilized by the starch matrix (Filek et al., 2010). Similarly, calcium (Ca) modification overturned the Cd stress-induced changes and increased the intercellular CO (2) concentration and NPQ as well as defied Cd accumulation in *B. napus* seedlings (Wan et al., 2011). In transgenic tobacco plants, cytokinin (CK) was shown to improve photosynthetic efficiency, cytochrome b6f (Cytb6f) complex formation, photosynthetic apparatus, and expression levels of genes associated with PSI and PSII as well as delay drought stress (Rivero et al., 2007, 2010). Figure 4 displays the complex hormonal crosstalk and regulation of stress-associated factors under stressful conditions. Thus, the studies on phytohormones provide fundamental background information about their potential crosstalk under stress conditions, but gene regulation and plant response could be more easily explained after the mutational studies, which will further disclose the comprehensive approaches and specific roles of different plant hormones in photosynthesis under both normal and stress conditions.

In addition to the analysis of individual phytohormones, some studies investigated the co-regulated hormonal responses to various stresses. For example, ABA and SLs together regulate carotenoid biosynthesis (Barickman et al., 2014), suggesting that the potential crosstalk between ABA and SLs (carotenoid-derived hormones) plays a role in the light-harvesting pathways. Recently, it was found that BR-dependent and GA-regulated transcriptome involved in cell wall formation and photosynthesis, the evidence indicates a strong association between various hormones and light-harvesting pathways (Attaran et al., 2014; Cortleven et al., 2014). Moreover, studies have reported that BRs modulate PSII efficiency and thylakoid architecture (Oh et al., 2011; Krumova et al., 2013; Dobrikova et al., 2014), which suggests that the coordinated crosstalk between BR and GA-signaling network regulates the adaptive response of plants to adverse environmental conditions.

Additionally, the coordination and crosstalk among phytohormone signaling networks somehow established the adaptive responses of plants under unfavorable conditions (Nishiyama et al., 2011; Ha et al., 2014). For example, abundance of ethylene during drought stress triggers leaf senescence and also disturbs ABA-mediated regulation of photosynthesis in leaf expansion (Bartoli et al., 2013). This suggests that the relation between ethylene and ABA and their relative abundance control the response behavior of plants to drought stress. Further studies are required to measure the effect of co-regulated hormones and points of intersection, which alter the regulation of the photosynthetic machinery or its components and reduce photoinhibition in chloroplasts under stress conditions. Moreover, we propose that additional genes and their roles in chloroplast should be identified to improve photosynthesis under abiotic stress.

## Effects on Activities of Key Photosynthetic Enzymes

The photosynthetic efficiency swiftly changes by various enzymatic reactions within cells during abiotic stresses (Kumar and Singh, 2009; Gill et al., 2011). The utmost noticeable effect of many stresses is the stomatal conductance and closure, which reduces the intercellular CO_2_ concentration by the deactivation of various enzymes such as Rubisco, sucrose-phosphate synthase (SPS), and nitrate reductase (Chaves et al., 2009; Mumm et al., 2011). Under high saline stress, the metabolic processes of photosynthesis are affected, and the thermostatic pressure is activated by a number of stroma enzymes that reduce CO_2_ (Xue et al., 2008; Biswal et al., 2011). Additionally, the activity of Rubisco is inhibited both *in vitro* and *in vivo* under high salt stress (Aragao et al., 2005). In general, the enhanced activity of Rubisco provides stability to plants under stress conditions, and the leaf rubisco level shows a positive relationship with phosphorous (P) and N levels in most C_3_ plants, except in glycophytic species; however, a contrasting relationship is detected under salt stress (Aragao et al., 2005; Taub, 2010; Makino, 2011). This contrasting equation needs further investigation to clarify the relationship between these traits. Ghosh et al. (2001) reported that under saline conditions in rice, two genotypes not only affect Rubisco but also its substrate, RuBP, which plays a key role in the Calvin cycle (Ghosh et al., 2001). Additionally, Pb affects CO_2_ fixation by altering RuBP and phosphoenol pyruvate carboxylase (PEPC) in the C3 and C4 cycles, respectively (Agarie et al., 2002; Häusler et al., 2002). Moreover, Pb also disturbs the activity of glyceraldehyde-3-phosphate dehydrogenase (GAPDH) and ribulose-5-phosphate kinase in the C3 cycle. Drought-responsive ABA hormone is mainly regulated by 9-cis-epoxy carotenoid dioxygenase (NCED) and d-arabino-1,4-lactone oxidase (ALO), enabling plant resistance (Bao et al., 2016). This is because ABA controls the balance between stomatal conductance and transpiration rate to prevent water loss under drought stress (Pirasteh-Anosheh et al., 2016). Moreover, some studies revealed that the photosynthetic efficiency remarkably reduced by fructose-1,6-bisphosphatase during stress. A significant reduction in the RuBP pool size has been reported in common bean (von Caemmerer and Farquhar, 1984), sunflower (Gimenez et al., 1992), and rice (Ghosh et al., 2001).

The activity of phosphoenolpyruvate carboxylase (PEPC) in wheat (C_3_ plant) and maize (C_4_ plant) is inhibited by salt stress, although the PEPC of wheat is less sensitive to salt stress than that of maize. Hirel et al. (2007) described the potential utility of the Rubisco content (i.e., improved WUE and yield components) in breeding programs (Hirel et al., 2007). Maize transgenic plants with enhanced expression of C_4_-PEPC showed 30% increase in WUE and 20% increase in dry biomass under water scarcity, which suggests that the maize transgene approach can be used as a tool improving the endogenous enzymes involved in the process of photosynthesis (Jeanneau et al., 2002). Previously, it was reported that the activities of the various C_4_ photosynthesis enzymes, such as PEPC, NADP-malic enzyme (NADP-ME), Rubisco and fructose-1,6-bisphosphatase were greatly limited under drought stress (Du et al., 1998). For example, the activity of pyruvate phosphate dikinase (PPDK) was reduced in C_4_ plants, which confirmed the role of PPDK in photosynthesis under drought conditions. Li et al. (2007) reported a reduction in the solubility of CO_2_ in the leaf tissue and regulation of the Rubisco enzyme at high temperatures, consequently reducing photosynthesis (Raines, 2011). Crafts-Brandner and Salvucci (2004) reported that Rubisco activity was significantly inhibited in both cotton and tobacco, when the leaf temperatures were exceeded at 35°C. A similar mechanism of the acclimation to high temperatures in C_4_ maize plants had showed association with manifestation of a larger subunit of Rubisco and limited recovery of the Rubisco activation state (Crafts-Brandner and Salvucci, 2002). This indicates that the decrease in Rubisco activation is highly affected by increase in temperatures. Chinthapalli et al. (2003) reported that PEPCase from the C_4_ plant (Amaranthus hypo-chondriacus) showed less sensitivity to supra-optimal temperatures and more sensitivity to sub-optimal temperatures as compared to the enzymes in C_3_ species (*Pisum sativum*) (Chinthapalli et al., 2003). It might suggest that the key photosynthetic enzymes in both C_3_ and C_4_ plants shows variation in sensitivity to higher temperatures.

Although all C_4_ plants exhibit greater tolerance to high temperature than C_3_ plants, the photosynthetic efficiency of C_4_ plants relatively sensitive to heat stress, probably due to the activation of Rubisco in C_4_ plants, which inhibits electron transport and PEP carboxylation/PEPC activity and regeneration. In the light of these findings, it can be concluded that the key photosynthetic enzymes in C_3_ and C_4_ plants exhibit different levels of sensitivity to temperature. Our conclusion is justified by Xu et al. (2003), who showed temperature-sensitive activity of photosynthesis related enzymes; in wheat plants under heat stress, Rubisco activity was reduced after 12 days, and PEPC activity remained unstable while the ratio of PEPC/Rubisco markedly increased. Enzymatic activities also reshape the photosynthetic pathways in C_3_ and C_4_ plants under various abiotic stresses, depending on several factors such as stomatal response, plant type, and interaction with enzymatic changes, thus altering the photosynthetic capacity. When plants are encountered by abiotic stresses, GAPDH converts glycerate-3-phosphate (G3P) to glyceraldehyde-3-phosphate, and the latter further promotes the ability to receive electrons from NADPH and rescue PSII from ROS (Hildebrandt et al., 2015). Previously, it was observed that GAPDH is up-regulated in wheat genotypes by PEG6000 treatment for 48 h, indicating that GAPDH plays a vital role in the maintenance of photosynthetic activity and promotion of plant growth (Cheng et al., 2015). Additionally, overexpression of the *GAPDH* gene in transgenic tobacco plants enhanced tolerance to drought stress (Kappachery et al., 2015). Another enzyme, ferredoxin-NADP reductase (FNR), helps to channelize electronic transport and redox homeostasis within chloroplasts (Chinthapalli et al., 2003). Different abiotic stresses affect the activity of FNR differently; for example, in transgenic tobacco plants, FNR level was reduced by drought stress (Gharechahi et al., 2015), whereas in *Paeonia cathayana* (Xiao et al., 2009), wheat (Budak et al., 2013), rice (Nouri et al., 2015; Chintakovid et al., 2017), and maize, FNR levels was increased by salt stress (Zörb et al., 2009). In rice, GAPDH activity was up-regulated, whereas FNR level was reduced under osmotic stress (Chintakovid et al., 2017). These outcomes indicate that different plant species employ different mechanisms to stabilize the electron flow during photosynthesis. Additionally, the activation of numerous enzymes is concerned with light, therefore, the light intensity and duration of intervals is another stress, that decide the activation, or deactivation of these enzymes. Additionally, enzymes are generally very sensitive their environment, and even small environmental fluctuations can affect their potential stability and functionality.

## Transcription Factors (TFs) and Their Association With Photosynthesis Under Stress Conditions

Gene expression is critically controlled by TFs that manipulate various cellular processes in almost all living organisms, although TF regulation for particular gene/genes is fundamentally dependent on the genome size of a given species. Hence, the main objective of researchers is to identify the molecular mechanism underlying gene expression and gain functional insights into the role of the specific gene/genes affecting specific traits control through regulation by TFs (Ashraf and Harris, 2013).

The function of various TFs involved in the regulation of photosynthesis related genes, either directly or indirectly (hormonal pathways), has been investigated previously (Saibo et al., 2009; Gururani et al., 2015). Plant TFs such as *BZR1* and *WRKY*, have been noticed to influence cell-wall and genes related to chloroplast, additionally, the efficiency of PSII system have shown correlation with the regulation of GhDREB and CRF6, and chlorophyll content (Waters et al., 2009; Nguyen et al., 2014). The regulation of *CAB* gene expression by the light-responsive LONG HYPOCOTYL 5 (HY5) and bZIP-type TFs plays a critical role in stress tolerance (Saibo et al., 2009). The CAB2 TF not only controls the expression of *Rubisco* but also regulates the expression of *RbcS1A* (a gene that encodes subunit 1A of Rubisco) (Maxwell et al., 2003; Lee et al., 2007). Similarly, the rice OsMYB4 TF is responsible for the accumulation of glycine betaine, which increases the adaptability of Arabidopsis plants under stress conditions. Moreover, glycine betaine improves the structure of Rubisco under saline conditions (Yang et al., 2005; Khafagy et al., 2009). In maize, the two TFs including DOF1 (activator) and DOF2 (repressor) regulate the expression of the *PEPC* gene under stress conditions (Yanagisawa and Sheen, 1998; Yanagisawa, 2000). Another study unveiled that CAM (Calmodulin)-specific genes in plants show high expression levels because of *cis*-acting and trans-acting transcriptional regulation under drought and salt stress conditions (Saibo et al., 2009). The MYB-type, Gap encoding NAD-dependent GAPDH, and Ppcl gene encoding a CAM-specific isozyme of PEPC regulation induced under saline stress and further activates photosynthesis-related genes (Schaeffer et al., 1995). Constitutive expression of *ABP9* and *bZIP* genes in transgenic Arabidopsis plants regulates pigment composition, photosynthetic carbon and ABA level in leaf tissue as well as activate after receiving stress signals (water deficit and heat stress) and involve in light harvesting conditions (Zhang et al., 2008). The C-repeat binding factor/dehydration-responsive element (CRT/DREB)-binding TF family controls the expression of key genes and sense environmental cues and actively coordinates signal transduction networks (Agarwal et al., 2017).

The expression of *CBF/DREB* genes respond to temperature fluctuations in Arabidopsis (Schramm et al., 2008; Demmig-Adams et al., 2018) as well as in other plant species (Akhtar et al., 2012; Kurepin et al., 2013; Kidokoro et al., 2015; Agarwal et al., 2017). The CBF/DREB TF family is divided into two groups; TFs in one group are primarily associated with cold stress adaptation, while those in the second group are predominantly involved in heat, drought, and salt adaptation (Demmig-Adams et al., 2018). The *CBF1, CBF2*, and *CBF3* genes, also known as DREB1B, C, and A, respectively, are dynamically involved in plant growth regulation under cold stress (Thomashow, 2010). *DREB2* TF genes are induced by drought and heat stress (Schramm et al., 2008; Chen et al., 2010), whereas *CBF4/DREB1D* and *DREB3* are induced by ABA treatment under drought stress (Haake et al., 2002; Shavrukov et al., 2016). Additionally, CBF/DREB1-type and DREB2-type TFs show overlapping functions and mechanized temperature adaptation strategies (Agarwal et al., 2017). Most importantly, CBF/DREB TFs regulate all co-regulated genes, which helps to maintain normal photosynthesis, stomatal conductance, Chl content, and ETC under stress conditions, thus improving plant growth and development.

Besides the well-identified role of TFs, some candidate genes also have been recognized as a fast and reliable way to directly link leaf photosynthesis in in crop plants with the level of tolerance to different abiotic stresses (Nawaz et al., 2018). Overexpression of the Arabidopsis *HARDY* (*HRD*) gene in rice enhanced photosynthetic assimilation and reduced the transpiration rate (Karaba et al., 2007). *ABR17*, a member of pathogenesis-related proteins (PR10), showed increased tolerance lto multiple stresses, and improved the rate of seed germination in Arabidopsis (Srivastava et al., 2004). The mitochondrial pentatricopeptide repeat (PPR) proteins play a vital role in post-transcriptional regulation and embryo development (Chintakovid et al., 2017). Further studies on the Arabidopsis *ppr40* mutant confirmed the importance of PPR proteins in organogenesis, embryo development, and irregular photosynthesis (Pusnik et al., 2007; Manna, 2015). The regulation of *PcINO1* and *McIMTI* genes in tobacco enhanced salt tolerance and within chloroplasts and cytosol increased the level of inositol, as well as transgenic plants endorse the growth and photosynthesis with minor oxidative damage compared with wild-type plants under salt stress (Patra et al., 2010). Mitogen-activated protein kinases (MAPKs) are involved in signal transduction from the chloroplast to the nucleus, chloroplast redox regulated gene expression, metabolic/cellular processes, and response to external stimulus in eukaryotes (Danquah et al., 2014; Dietz et al., 2016; Raja et al., 2017). Constitutive expression of the tobacco *NPK1* gene in maize enhanced drought tolerance (Zong et al., 2009) and alleviated the rate of photosynthesis under water deficit stress (Zhang et al., 2012). Similarly, sugar-mediated regulation of genes enhanced the photosynthetic components in sugar deficient situation (Pego et al., 1999), whereas the regulation of genes related to sugar content enhanced the photosynthetic capacity by increasing the level of sugar in leaf, thus activating the photosynthetic components and increasing photosynthesis (Pego et al., 2000). Together, these studies suggest that the regulation of specific genes improves the processes of photosynthesis or photosynthesis related machinery.

## Biodegradable and Biostimulant Compounds Boost Plant Health and Significantly Improve the Process of Photosynthesis

Excess application of inorganic fertilizers pollutes the environment and probably provokes natural disasters. Therefore, to avoid or escape from this situation, the research interest has shifted to exploring advanced strategies employing biodegradable compounds such as chitosan (CT), beneficial fungi and humic acid (HA) as well as biostimulants (melatonin and bio-waste) to increase plant growth, photosynthesis, nutrition value, soil structure/texture, and stress tolerance (Castro et al., 2012; Petrozza et al., 2014; Bulgari et al., 2015; du Jardin, 2015; Saa et al., 2015).

CT is produced from chitin, a basic constituent of sea food shells, and shows great application potential, especially under stress conditions (Sharif et al., 2018). CTs promote the seed germination rate and facilitate plant nutrient uptake in wheat under salt stress (Zong et al., 2017; Li et al., 2019). A recent study showed that CT biopolymers efficiently improve plant physiology and gene regulation, and facilitate the activation of plant defense signaling pathways to increase drought stress tolerance (Sharif et al., 2018). Similarly, spray application of CTs enhanced plant growth, Chl content, and photosynthetic machinery in *B. napus* plants under Cd stress (Zong et al., 2017). Furthermore, CTs induced ABA activity, which plays an imperative role in the regulation of stomatal aperture to lower the transpiration rate under stress conditions (Turk, 2019). Foliar application of CTs (250 mg/L) increased the amount of Chl and total carbohydrates in cowpea (Farouk and Amany, 2012); the same affect was observed in maize, soybean and bean using chitin oligosaccharides (Hidangmayum et al., 2019; Mukhtar Ahmed et al., 2020). Therefore, it can be speculated that might increase in N and K content boost chloroplast cells in shoot, that further enhance Chl synthesis. The aforementioned reports shed light on the beneficial effect of CTs on plant health and photosynthetic machinery under stress conditions.

HA is an organic fertilizer derived from organic waste materials that can enhance plant growth and development. Foliar application of HA markedly increases photosynthesis and Chl content and improves the chloroplast ultrastructure and other morphological features in chrysanthemum (*Chrysanthemum morifolium*) seedlings (Fan et al., 2014). In *Plantago ovata*, application of HA and fulvic acid resulted in noticeable effects on Chl content and photosynthesis under salt stress (Gholami et al., 2013). Similarly, in common bean the HA supplements shoot-up plant morphology, biomass, and Chl content under salt stress (Meganid et al., 2015).

Melatonin (N-acetyl-5-methoxy tryptamine) is a ubiquitous molecule and most probably synthesized in chloroplasts and mitochondria and then translocated to other plant parts (Arnao and Hernández-Ruiz, 2014, 2019; Kołodziejczyk and Posmyk, 2016; Sun et al., 2020). In plants, HA performs various biological functions, such as signaling molecule stimulation, photo response regulation, stress tolerance, and circadian rhythm (Reiter et al., 2015; Hu et al., 2016; Nawaz et al., 2016). Melatonin participates in signal transduction and improvement of the photosynthetic machinery in several plant species (Table 2), exogenous application of melatonin induces physiological modifications and resistance against several abiotic stresses (Xu et al., 2016a; Liang et al., 2018). For example, melatonin application enhances high temperature tolerance in cucumber seedlings (Xu et al., 2010), cold resistance in tomato (Zhang et al., 2021), salt tolerance in rice (Liang et al., 2015) and vanadium (V), and salt stress tolerance in watermelon (*Citrullus lanatus*) (Li Z. et al., 2017; Nawaz et al., 2018). In rice seedlings, melatonin delayed leaf senescence by improving the Chl content of leaves, which reduced the level of ROS either directly or indirectly and promoted antioxidant activity (Liang et al., 2015). Similarly, pretreatment of watermelon seeds with melatonin enhanced photosynthate assimilation, Chl content, ROS production, and antioxidant activity in watermelon seedlings (Li Z. et al., 2017; Nawaz et al., 2018). Melatonin biosynthesis increases the tolerance to salt stress by improving chloroplast structure and the process of photosynthesis (Zheng et al., 2017). Moreover, melatonin protects against drought stress by protecting the chloroplast ultrastructure in the spongy mesophyll of grape leaves, thereby improving the efficiency of PSII (Meng et al., 2014). Table 2 listed the effect of melatonin on photosynthesis in several crops.

**Table 2:** Induced photosynthetic activity and growth attributes by melatonin in various crops under abiotic stresses.

**Specie**	**Stress**	**Concentration**	**Functions**
Arabidopsis	Heat	1,000 μM	Delayed leaf senescence and maintained growth (Hernández et al., 2015)
Apple	Drought	100 μM	Enhance ABA activity and radical scavenging (Li et al., 2014)
Alfalfa	Drought	10 μmol/L	Increase proline metabolism (Antoniou et al., 2017)
Cucumber	Salinity	100 μM	Overall growth (Wang et al., 2016)
Grapes	Water	200 μmol/L	Improved antioxidative enzymes activity (Meng et al., 2014)
	deficient		
Maize	Drought	100 μmol/L	Photosynthesis and growth (Ye et al., 2016)
Perennial ryegrass	High temperature	20 μM	Regulate abscisic acid and cytokinin biosynthesis (Zhang et al., 2017)
Red cabbage	Heavy metal	10 μM	Improved seed germination and reduce the toxic effect of metal in seedling (Posmyk et al., 2008)
Soybean	Multiple stress	100 μM	Boost and maintain the overall plant growth (Wei et al., 2014)
Tomato	Cold and salinity	100 μM	Improved photosynthesis and regulation of photosynthetic electron transport (Zhou et al., 2016; Yang et al., 2018)
Watermelon	Salinity	150 μM	Redox homeostasis and improved photosynthetic activity (Li H. et al., 2017)
Wheat	Drought and metal	500 μM and 1 mM	Increased seedling percentage, growth, and antioxidant enzymes activities (Cui et al., 2017; Zuo et al., 2017)

Despite these biostimulant applications, melatonin had prominent role in transgenic crops such as the N-acetylserotonin-O-methyltransferase (ASMT) which is a specific enzyme required for melatonin synthesis. *MzASMT1* from apple rootstock (*Malus zumi* Mats) was induced by drought stress in apple leaves, while its over-expression in transgenic *Arabidopsis*, the melatonin levels were 2–4 times higher than those in the wild type which indicates a positive relation of *MzASMT1* to melatonin production in drought stress (Zuo et al., 2014). The apple SNAT (serotonin N-acetyltransferase, *MzSNAT5*) was highly expressed by drought in transgenic *Arabidopsis* and elevated the melatonin levels thus enhanced tolerance to drought (Wang L. et al., 2017). Likewise, wheat *TaCOMT* (Caffeic acid 3-O-methyltransferase), gene over-expression enhances drought tolerance and produced higher melatonin, proline, and lower malondialdehyde (MDA) contents, than that in wild type (WT) plants in transgenic *Arabidopsis* (Yang et al., 2019).

Overexpression of N-acetyltransferase1 and human serotonin N-acetyltransferase in transgenic rice confirmed resistance against cold and Cd stress (Kang et al., 2007), while overexpression of alfalfa *SNAT* in Arabidopsis conferred greater resistance to salt stress and increased reestablishment of redox and ion homeostasis compared with the wild type (Zhao et al., 2019). Moreover, expression of ovine *AANAT* and *HIOMT* genes in switch grass (*Panicum virgatum*) improved plant growth and salt tolerance (Huang et al., 2017). However, we still covered the limited application of melatonin in the improvement of photosynthesis under abiotic stresses, but in the light of current contribution of melatonin proved significant clues of photosynthesis and plant growth improvements. Although, we suggest further investigation to identify specific genes regulated by biodegradable and biostimulant treatments that improve the process of photosynthesis.

## Conclusion and Future Perspectives

In the current review article, we summarized the research progress made so far in the field of photosynthetic performance under abiotic stresses and several approaches that have been successfully used to increase the productivity and abiotic stress tolerance of plants, although many other challenging ideas are yet to be explored.

Abiotic stresses have a common mode of toxicity in plants which is the production of ROS, causing oxidative damage, and membrane instability. Therefore, genetic manipulation of antioxidants, sufficient knowledge of ROS signaling and its regulatory responses, stress regulatory pathways, and functional characterization of key genes can increase tolerance to a variety of stresses. Although numerous studies have thoroughly discussed the significance of genes involved in abiotic stress, but the specific role in photosynthetic machinery still needs deep investigation. Moreover, approaches toward the improvement of the components of PSII and LHC should be shifted toward the ROS scavenging and antioxidant activity. Additionally, the elucidation of redox signaling pathways related to both ROS and antioxidants could provide much more useful molecular tools for the up-regulation of whole suites of genes with protective functions that could make the photosynthetic system much less susceptible to the light- and ROS-mediated modifications.

Evidence shows that stresses usually depends upon their intensity and duration that either up-regulate or down-regulate the genes involved in the mechanism of photosynthesis in plants. Thus, the obtaining the expression patterns of such genes can help to understand the plant photosynthetic or other metabolic responses to various stresses and further functional validation may enhance the photosynthetic capacity in different crops under stressful conditions. We propose that a series of comprehensive and more logical studies need to be conducted that cover the genomic to proteomic and physiological to biochemical analyses of plants under stress. The transgenic approaches still carry a larger scope for a better crop improvement, where the diverse range of abiotic stresses needed to be tested on the basis of the inducible promoter targeting a particular tissue, stage, and specific environmental stress, this may allow to grow transgenic plants under harsh environment with minimum yield losses as well as help in understating the genetic and environmental interactions.

Moreover, the alteration of photosynthesis by phytohormones under stress is not based on a single event but on a complex process of signaling networks and the expression behavior of phytohormone regulated photosynthetic genes. Here, the putative functions of genes during photosynthesis and the correlation between transcriptomic profiling and phytohormone regulatory networks largely link the valuable data set. It would be helpful to further explain the cellular response to environmental stresses and the transcriptional regulation of photosynthetic metabolism rate.

The redox state is regulated by various environmental factors, and the destabilized redox state reprograms the process of photosynthetic ETC, chloroplast genes and various other metabolic, and signaling responses. Therefore, in future research, the efficiency of light use and photosynthetic electron transport yield, improving carbon assimilation, and limiting its loss during photorespiration are the main points to be addressed. Previously, documented data indicated that photosynthesis efficiency decline by the inhibition of RUBISCO activity, therefore, we suggest that modifications of Rubisco to obtain higher turnover rates where the substrate specificity is not a very promising direction for future research. It would be more useful to focus on three approaches, to improve carbon fixation via genetic engineering, namely improving the catalytic properties of Rubisco, introduction of carbon concentration mechanisms (CCMs) and engineering of photorespiration. This would improve the Rubisco and increase the rate of the RuBP regeneration phase in chloroplast. Although TFs play a major role in abiotic stress responses, the regulation of photosynthetic genes by TFs is not yet fully understood. In addition, the area should be investigated more thoroughly in terms of their significance for photosynthesis improvement, plant productivity, and stress resistance. Overall, data suggests that the enhanced photosynthetic machinery and metabolic signaling with improved stress tolerance in plants necessitate a more comprehensive understanding of the inter-disciplinary strategies based on different areas of research.

## Author Contributions

IM conceived the idea, designed the outlines, and wrote the manuscript. AS and MA revised the initial draft. Q-HY and HA revised the final manuscript. FL provided useful ideas and funding. All authors approved the final version of manuscript.

## Conflict of Interest

The authors declare that the research was conducted in the absence of any commercial or financial relationships that could be construed as a potential conflict of interest.
